# Single Arginine Mutation in Two Yeast Isocitrate Dehydrogenases: Biochemical Characterization and Functional Implication

**DOI:** 10.1371/journal.pone.0115025

**Published:** 2014-12-11

**Authors:** Ping Song, Huanhuan Wei, Zhengyu Cao, Peng Wang, Guoping Zhu

**Affiliations:** Institute of Molecular Biology and Biotechnology, Anhui Normal University, No. 1 Beijing East Road, Wuhu, 241000, Anhui, China; United States Army Medical Research Institute of Infectious Diseases, United States of America

## Abstract

Isocitrate dehydrogenase (IDH), a housekeeping gene, has drawn the attention of cancer experts. Mutation of the catalytic Arg132 residue of human IDH1 (HcIDH) eliminates the enzyme's wild-type isocitrate oxidation activity, but confer the mutant an ability of reducing α-ketoglutarate (α-KG) to 2-hydroxyglutarate (2-HG). To examine whether an analogous mutation in IDHs of other eukaryotes could cause similar effects, two yeast mitochondrial IDHs, *Saccharomyces cerevisiae* NADP^+^-IDH1 (ScIDH1) and *Yarrowia lipolytica* NADP^+^-IDH (YlIDH), were studied. The analogous Arg residues (Arg148 of ScIDH1 and Arg141 of YlIDH) were mutated to His. The *K*_m_ values of ScIDH1 R148H and YlIDH R141H for isocitrate were determined to be 2.4-fold and 2.2-fold higher, respectively, than those of the corresponding wild-type enzymes. The catalytic efficiencies (*k*_cat_/*K*_m_) of ScIDH1 R148H and YlIDH R141H for isocitrate oxidation were drastically reduced by 227-fold and 460-fold, respectively, of those of the wild-type enzymes. As expected, both ScIDH1 R148H and YlIDH R141H acquired the neomorphic activity of catalyzing α-KG to 2-HG, and the generation of 2-HG was confirmed using gas chromatography/time of flight-mass spectrometry (GC/TOF-MS). Kinetic analysis showed that ScIDH1 R148H and YlIDH R141H displayed 5.2-fold and 3.3-fold higher affinities, respectively, for α-KG than the HcIDH R132H mutant. The catalytic efficiencies of ScIDH1 R148H and YlIDH R141H for α-KG were 5.5-fold and 4.5-fold, respectively, of that of the HcIDH R132H mutant. Since the HcIDH Arg132 mutation is associated with the tumorigenesis, this study provides fundamental information for further research on the physiological role of this IDH mutation *in vivo* using yeast.

## Introduction

Isocitrate dehydrogenase (IDH) is an enzyme of the tricarboxylic acid (TCA) cycle and catalyzes the oxidative, NAD(P)^+^-dependent dehydrogenation and decarboxylation of isocitrate to α-ketoglutarate (α-KG) and CO_2_. Due to its critical metabolic function in the cell, IDH is found in organism from all domains of life. IDH can be subdivided according to its cofactor specificity into NAD^+^-and NADP^+^-specific IDHs. Eukaryotes usually have both types of IDH. Eukaryotic NAD^+^-IDH always localizes in the mitochondria and participates in energy metabolism, whereas NADP^+^-IDHs are distributed throughout different cell compartments and function in diverse cellular processes.

The most extensively investigated eukaryotic IDHs are those from human and yeast. Three distinct IDH isoforms are found in human cells. Both cytosolic NADP^+^-IDH (IDH1) and mitochondrial NADP^+^-IDH (IDH2) are homodimers, whereas the mitochondrial NAD^+^-IDH (IDH3) is a heterooctamer [1], [2]. In addition to their potential catabolic role in the TCA cycle, both IDH1 and IDH2have been demonstrated to aid in the cellular defense against oxidative damage by generating NADPH [3]–[5]. In *Saccharomyces cerevisiae*, there are three homologous NADP^+^-IDHs: mitochondrial NADP^+^-IDH1 (ScIDH1), cytosolic NADP^+^-IDH2 (ScIDH2), and peroxisomal NADP^+^-IDH3 (ScIDH3) [6]–[10]. Each of these proteins plays distinct roles in the central metabolism of *S. cerevisiae*: ScIDH1 is involved in the cellular glutamate synthesis, ScIDH2 provides NADPH for thiol-based antioxidant systems, and ScIDH3 generates NADPH for the β-oxidation of unsaturated fatty acids [10]. In *Yarrowia lipolytica*, a 'non-conventional' species of oleaginous yeast, has only one NADP^+^-IDH (YlIDH), which is located in the mitochondria and is closely related to the oleaginicity of *Y.lipolytica* [11].

As a fundamental metabolic enzyme, IDH has been extensively studied both kinetically and structurally. However, few new studies on IDH have been published in recent years. Furthermore, interest in human IDH1 and IDH2 in tumor metabolism studies has recently increased since the publication of a cancer genome project showing that the genes encoding IDH1and IDH2 were mutated in glioblastoma multiforme (GBM), in 2008 [12]. The scientific community was further encouraged to consider this abnormality when another cancer genome project also reported the very same mutation in IDH1 and IDH2 of acute myeloid leukemia (AML) patients [13].

**Figure 1:**
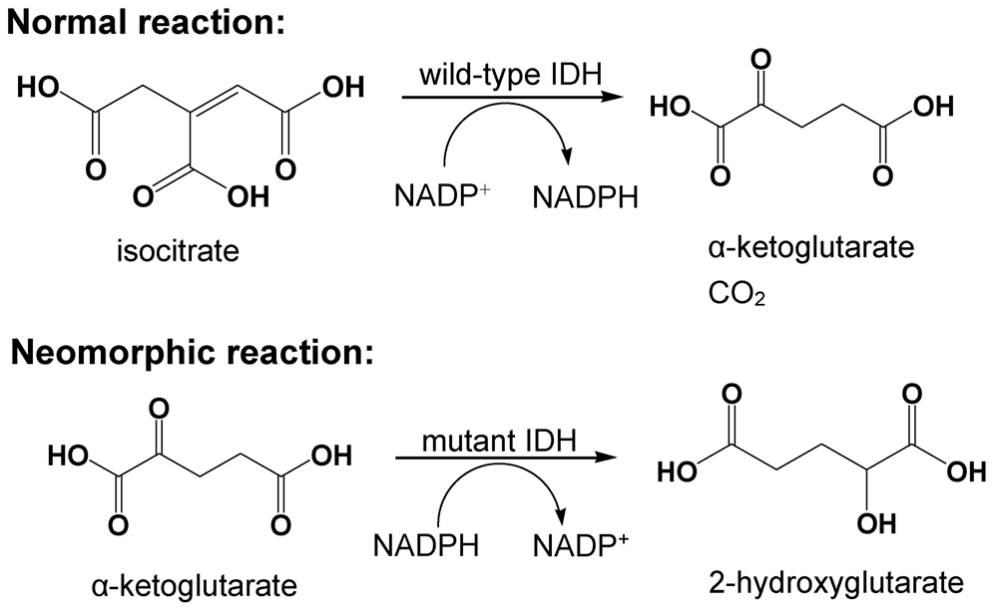
Normal and neomorphic reactions catalyzed by wild-type and mutant IDH. The normal reaction converts isocitrate to α-ketoglutarate and CO_2_ accompanying with NADP^+^ reduction, while the neomorphic reaction transforms α-ketoglutarate to 2-hydroxyglutarate in an NADPH dependent manner.

Recently, human IDH mutations have been reported to be somatic and monoallelic and to occur at a single residue located in the substrate-binding pocket. Mutations in IDH1 of GBM patient almost always affect arginine 132 (R132) [12], which has been found to be converted to histidine, serine, cysteine, glycine or leucine, with histidine (R132H) being the most frequent change [14]. GBM-associated IDH2mutations are present at the homologous R172 residue (with the alterations being predominantly R172K), and AML-associated mutations are found at another active site, arginine R140 (with the alterations being predominantly R140Q) [14], [15]. As R132 and R172 are hotspots in the substrate-binding site of IDH1 and IDH2 [1], mutations affecting these amino acids lead to structural alteration of the active site of the enzyme, resulting in the simultaneous loss of their normal catalytic activity and the production of α-ketoglutarate (α-KG) and NADPH [16]. However, mutant IDH enzymes acquire a neomorphic activity in which the normal product, α-KG, is reduced to 2-hydroxyglutarate (2-HG) while NADPH is oxidized to NADP^+^ [15], [17]–[19] (Fig. 1). 2-HG is structurally similar to α-KG and acts as α-KG antagonist to inhibit multiple α-KG-dependent enzymes involved in epigenetic regulation, collagen synthesis, and cell signaling [20]–[22]. The two most recent studies confirmed that *in vivo*, IDH2 mutations inhibit differentiation, alter DNA methylation and result in hyper proliferation in diverse tumor contexts. Furthermore, IDH2 is required for tumor maintenance rather than simply for tumor initiation [23], [24].

The R132 residue of IDH1 is completely conserved at the corresponding sites in all bacterial and eukaryotic NADP^+^-IDH enzymes [1], which implies that the analogous mutation would transform all homologous IDHs into new enzymes and would result in the neoenzymatic activity of α-KG to 2-HG conversion. In the present study, we studied two yeast NADP^+^-IDHs, *S. cerevisiae* mitochondrial NADP^+^-IDH1 (ScIDH1) and *Y. lipolytica* mitochondrial NADP^+^-IDH (YlIDH). Both of the analogous Arg residues of human IDH, Arg132 and Arg148 of ScIDH1 and Arg141 of YlIDH, were mutated to His. Detailed assays were performed to assess the enzymatic characteristics of the wild-type and mutant IDHs. We demonstrated for the first time that alteration of the analogous Arg to His confers NADP^+^-IDHs from the two lower eukaryotes the ability to convert α-KG to 2-HG with moderate catalytic efficiency. Because the HcIDH Arg132 mutation is associated with tumorigenesis, it will be interesting to investigate the physiological role of the mutated IDHs in yeast cells. This study provides fundamental information for further research on the physiological role of this IDH mutation *in vivo* using yeast.

## Materials and Methods

### Vectors and reagents

The plasmid pET-28b(+) (Novagen, USA) was used to carry the target gene. PrimeSTAR HS DNA polymerase was purchased from TaKaRa (Dalian, China). Restriction enzymes and protein molecular weight standards were obtained from Fermentas (Shanghai, China). Proteins were purified using BD TALON Metal Affinity Resin (Clontech, USA). NADH and NAD(P)H were obtained from Bio Basic Inc. (Canada).N-methyl-N trimethyl silicon alkyl-three fluoroacetamide (MSTFA), trimethylchlorosilane (TMCS) and methoxamine hydrochloride were purchased from Sigma (USA).

### Recombinant plasmid construction

Genomic DNA was extracted from *S. cerevisiae* S288c and *Y. lipolytica* CLIB122. The complete *HcIDH* gene was ordered from company. The PCR products of *ScIDH1*, *YlIDH* and *HcIDH* were digested with corresponding restriction enzymes and ligated into expression vector pET-28b(+) to generate the recombinant expression vectors pET-*ScIDH1*, pET-*YlIDH* and pET-*HcIDH*. Primers used in this study were listed in the Table 1.

**Table 1:** Primers used in this study.

Primers	Nucleotide sequences 5′→3′
ScIDH1-S^a^	CCACCTCTCGCCTTCATATGTTCAGTAAGATTAAGGTCAAAC
ScIDH1-As^a^	CCGCTCGAGTTACTCGATCGACTTGATTTCTTTTTGTAG
YlIDH-S	GGAATTCCATATGTCCACCACCGCTACTCGAG
YlIDH-As	TATCAAATGCGGCCGCCTAAGCCAGGTCCTTCTTCAGT
HcIDH-S	GGAATTCCATATGTCTAAGAAAATCTCTGGCGGTAGCG
HcIDH-As	CCGCTCGAGCAGTTTAGCCTGAGCCAGTTTAATCT
ScIDH1 R148H-f^b^	CAATCATTATTGGACACCACGCCCACGGTGATC
ScIDH1 R148H-r^b^	GATCACCGTGGGCGTGGTGTCCAATAATGATTG
ScIDH1 R148A-f	CAATCATTATTGGAGCCCACGCCCACGGTGATC
ScIDH1 R148A-r	GATCACCGTGGGCGTGGGCTCCAATAATGATTG
ScIDH1 R148E-f	CAATCATTATTGGAGAACACGCCCACGGTGATC
ScIDH1 R148E-r	GATCACCGTGGGCGTGTTCTCCAATAATGATTG
YlIDH R141H-f	GCCTATCATCATTGGTCACCACGCCCACGGCGACCAG
YlIDH R141H-r	CTGGTCGCCGTGGGCGTGGTGACCAATGATGATAGGC
YlIDH R141A-f	GCCTATCATCATTGGTGCCCACGCCCACGGCGACCAG
YlIDH R141A-r	CTGGTCGCCGTGGGCGTGGGCACCAATGATGATAGGC
YlIDH R141E-f	GCCTATCATCATTGGTGAGCACGCCCACGGCGACCAG
YlIDH R141E-r	CTGGTCGCCGTGGGCGTGCTCACCAATGATGATAGGC
HcIDH R132H-f	GATCATCATCGGTCACCACGCGTACGGCGAC
HcIDH R132H-r	TCGCCGTACGCGTGGTGACCGATGATGATC
HcIDH R132A-f	GATCATCATCGGTGCGCACGCGTACGGCGAC
HcIDH R132A-r	TCGCCGTACGCGTGCGCACCGATGATGATC
HcIDH R132E-f	GATCATCATCGGTGAACACGCGTACGGCGAC
HcIDH R132E-r	TCGCCGTACGCGTGTTCACCGATGATGATC

a “-S” and “-As”: indicate the sense (-S) and antisense (-As) primers of the corresponding genes.

b “-f” and “-r”: indicate the forward (-f) and reverse (-r) primers used in the site-directed mutagenesis.

Underlined bases indicate the mutant sites.

### Sequence analysis

The amino acid sequences of IDH were downloaded from GenBank via the NCBI website (http://www.ncbi.nlm.nih.gov/). The X-ray structures of human cytosolic (HcIDH, 1T0L) and yeast mitochondrial IDH (ScIDH1, 2QFV) were downloaded from the PDB database (http://www.rcsb.org/pdb/). Amino acid sequence alignment was conducted using the ClustalX program (ftp://ftp.ebi.ac.uk/pub/software/clustalw2) and structure-based alignment was performed using ESPript 2.2 web tool (http://espript.ibcp.fr/ESPript/ESPript/) [25], [26].

### Site-directed mutagenesis

Arg148 (R148H, R148A and R148E), Arg141 (R141H, R141A and R141E) and Arg 132 (R132H, R132A and R132E) point mutations were introduced into ScIDH1, YlIDH and HcIDH, respectively, by overlap extension PCR-based site-directed mutagenesis. The overlap mutant primer sets were listed in Table 1. The PCR products for all the mutants were digested with the corresponding restriction enzymes and ligated into the expression vector pET-28b(+) to generate the recombinant expression vectors.

### Expression and purification of the wild-type and mutant enzymes

The wild type and mutant forms of ScIDH1, YlIDH and HcIDH were purified from *E. coli* Rosetta (DE3) clones over-expressing these proteins. The transformed cells were cultured overnight at 37°C in Luria-Bertani (LB) medium containing 30 µg/mL kanamycin and 25 µg/mL chloramphenicol. The cells were then inoculated (1∶100) into 50 mL of fresh LB medium containing the same antibiotics and grown in 250 mL flasks at 225 rpm and 37°C until the culture density reached an OD_600_ of 0.5–0.6. Isopropyl-β-D-1-thiogalactopyranoside (IPTG) was added to the culture at a final concentration of 0.1 mM with subsequent cultivation for 20 h at 20°C.

The cells were harvested by centrifugation at 4,000 rpm for 5 min at 4°C and resuspended in equilibration/wash buffer (50 mM sodium phosphate, 300 mM NaCl, pH 8.0). After sonication, the cell debris was removed by centrifugation at 11,000 rpm for 20 min at 4°C. The 6×His-tagged wild-type and the mutant enzymes of YlIDH, ScIDH1 and HcIDH were purified using BD TALON Metal Affinity Resin according to the manufacturer's instructions on ice. The purity of the enzymes was determined by SDS-polyacrylamide gel electrophoresis (SDS-PAGE), and the proteins were stained with Coomassie Brilliant Blue R-250.

### Circular dichroism spectroscopy of the wild-type and mutant enzymes

Circular dichroism (CD) spectroscopy was performed using a Jasco model J-810 spectropolarimeter. The ellipticity measurements as a function of the wavelength were performed as described previously [27]. Purified protein samples (0.2 mg/mL) were prepared in 20 mM sodium phosphate and 75 mM Na_2_SO_4_ (pH 8.0), and the ellipticity (*θ*) was obtained by averaging 3 scans of the enzyme solution between 195 and 260 nm at 0.5-nm increments. The mean molar ellipticity, [*θ*] (deg cm^2^ dmole^−1^), was calculated using the relationship [*θ*] = *θ*/10nCl, where *θ* is the measured ellipticity (millidegrees), *C* is the molar concentration of the protein, *l* is the cell path length in centimeters (0.1 cm), and *n* is the number of residues per subunit of enzyme (419 for wild-type and mutant ScIDH1; 422 for wild-type and mutant YlIDH; 420 for wild-type and mutant HcIDH).

### Enzyme assay

The isocitrate oxidation activities of wild-type and mutants of ScIDH1, YlIDH and HcIDH were determined spectrophotometrically by measuring the reduction of NADP^+^ to NADPH at 340 nm. The standard assay solution contained 50 mM Tris-HCl buffer (pH 8.0), 2 mM MgCl_2_, 1 mM DL-isocitrate, and 0.5 mM NADP^+^ in a 1 mL volume at 25°C. The increase in NADPH was monitored at 340 nm using a thermostated Cary 300 UV-Vis spectrophotometer (VARIAN, USA). The activities were calculated using a molar extinction coefficient of 6220 M^−1^cm^−1^. One unit (U) of enzyme activity was defined as the amount of enzyme that catalyzed the formation of 1 µmol of NADPH per minute under the standard assay conditions. The reaction was initiated by adding the wild-type or mutant enzyme at the appropriate concentrations. To determine the *K*_m_ of isocitrate, the concentration of isocitrate was varied, where as the standard concentrations of NADP^+^ and Mg^2+^ were held constant. The apparent kinetic parameters were obtained by nonlinear regression using the program Prism 5.0 (Prism, GraphPad Software, CA, USA), except that the *K*_m_ values of isocitrate of ScIDH1 R148A, ScIDH1 R148E, YlIDH R141A, HcIDH R132H and HcIDH R132A mutants were obtained by linear regression because the weaker affinity for isocitrate.

The α-KG reduction activities of ScIDH1 R148H, YlIDH R141H and HcIDH R132H were assayed by measuring the conversion of NADPH to NADP^+^. ScIDH1 R148H or YlIDH R141H and or HcIDH R132H was added to 1 mL of an assay solution consisting of 50 mM Tris-HCl (pH 8.0), 2 mM MgCl_2_, 0.2 mM NADPH, and 0.2 mM α-KG, and the decrease of absorbance at 340 nm over time was measured. All kinetic parameters were determined in at least three independent experiments. The protein concentrations were determined using the Bio-Rad protein assay kit (Bio-Rad, USA) with bovine serum albumin as a standard.

### Gas chromatography/time of flight-mass spectrometer (GC/TOF-MS) analysis

Assay solutions consisting of 50 mM Tris-HCl (pH 8.0), 2 mM MgCl_2_, 0.2 mM α-KG, 0.2 mM NADPH and the appropriate amount of purified mutant enzymes (ScIDH1 R148H and YlIDH R141H) in a 1 mL volume were incubated in a water bath for 2 hours at 25°C. The reaction mixture was then collected using a Millipore protein concentration kit (USA). The mixture was dried under nitrogen gas, oximated with 40 µL of a 20 mg/mL pyridine methoxyl amine solution at 30°C for 90 min, and then silanizated at 37°C for 30 min using a 40 µL solution of MSTFA and TMCS at a ratio of 99∶1 (vol/vol).

The supernatant after derivatization was then analyzed using a GC/TOF-MS system (gas chromatography HP6890, Agilent, USA and time of flight-mass spectrometer, Waters, USA), with a DB-5 chromatographic column (30 m×0.25 mm id, film thickness 0.25 µm, Agilent, USA). The temperature of the column was initially set at 70°C and was then increased by 5°C/min to 300°C. The solvent was delayed for 5 min, a 1 µL sample was injected at a constant velocity at 260°C, and the velocity of the Helium developing solvent was 1 mL/min. The temperature of the ion source was 220°C and the temperature of the union lever was 220°C. The ionizing voltage was 70 eV, and the scanning range of the mass spectrum was between 50 and 800 (m/z).

### Ethical statement

This study was a novel investigation of two yeast isocitrate dehydrogenases, and no human subjects material or data were directly involved (the complete *HcIDH* gene was obtained through chemical synthesis from biotechnology company). The human data utilized for comparison were all cited from published articles and are appropriately marked in the manuscript.

## Results and Discussion

### Bioinformatics analysis

**Figure 2:**
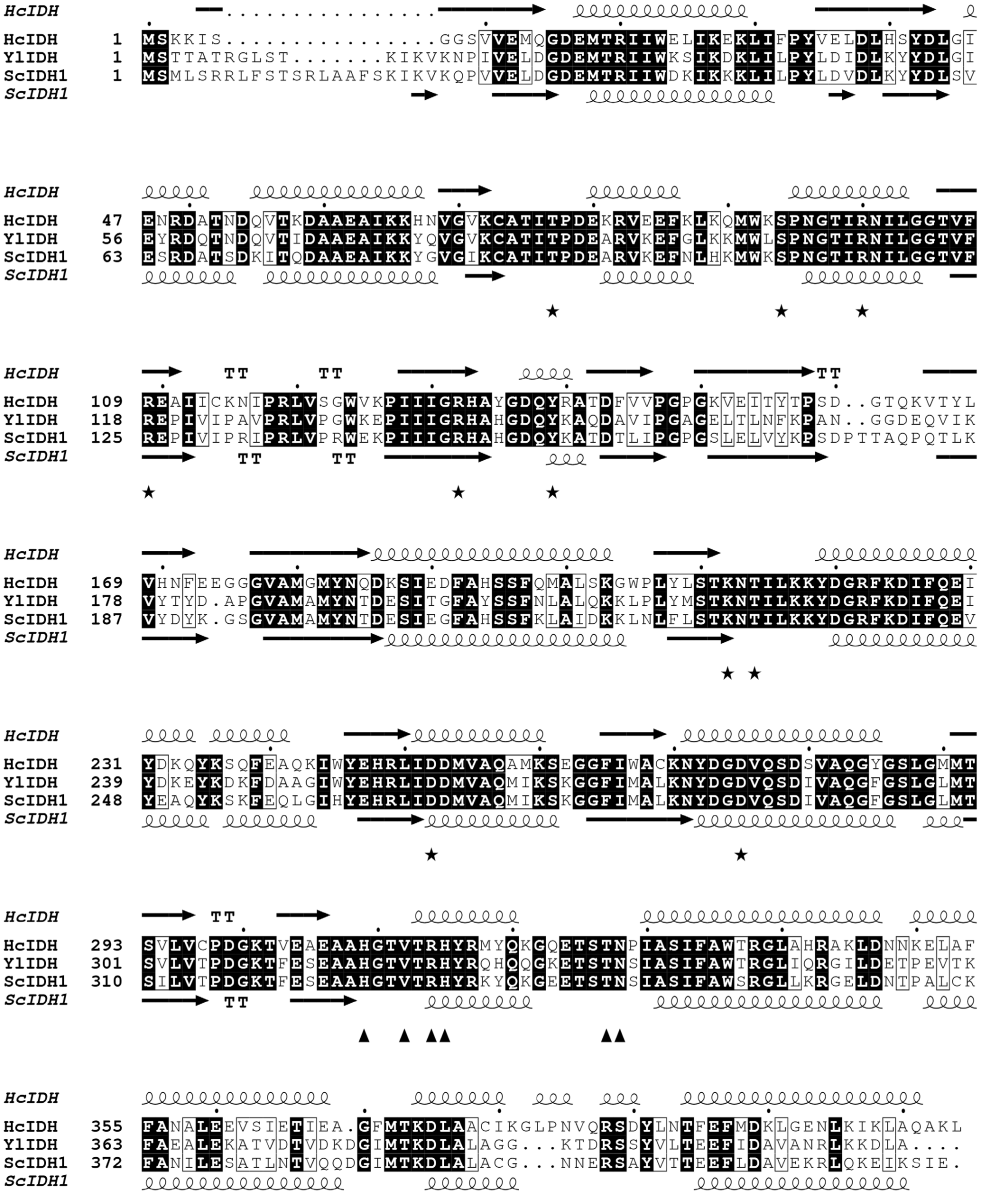
Structure-based protein sequence alignment of ScIDH1, YlIDH and HcIDH. The conserved amino acid residues are shaded. The secondary structure components, j.e, α-helices and β-sheets, of HcIDH and ScIDH1 are also shown. The conserved amino acid residues involved in substrate binding (★) and cofactor binding (▴) are indicated. This figure was generated using ESPript 2.2.

The sequence comparison between the two NADP^+^-IDH from lower eukaryotes, ScIDH1 and YlIDH, and human IDH1 (HcIDH) is presented in Fig. 2. The sequence identity among these three eukaryotic IDHs is greater than 60%, suggesting their comparable positions in evolution. As shown in Fig. 2, Arg132 of HcIDH is an evolutionarily invariant residue in NADP^+^-IDHs. Some other critical residues, such as Tyr139 and Lys212 that are important for isocitrate binding and catalysis in wild-type HcIDH and for α-KG binding and catalysis in the mutant HcIDH are also conserved in ScIDH1 and YlIDH (Fig. 2). The crystal structures of HcIDH and ScIDH1 have been solved [1], [9], and the secondary structural elements are shown in Fig. 2. The overall three-dimensional structures of HcIDH and ScIDH1 are very similar and consequently share an equivalent isocitrate catalysis mechanism, indicating the possibility that the analogous mutation will also confer to ScIDH1 and YlIDH a similar neomorphic ability as that of HcIDH (i.e., the conversion of α-KG to 2-HG).

### Overexpression and purification of the wild-type and mutant enzymes

**Figure 3:**
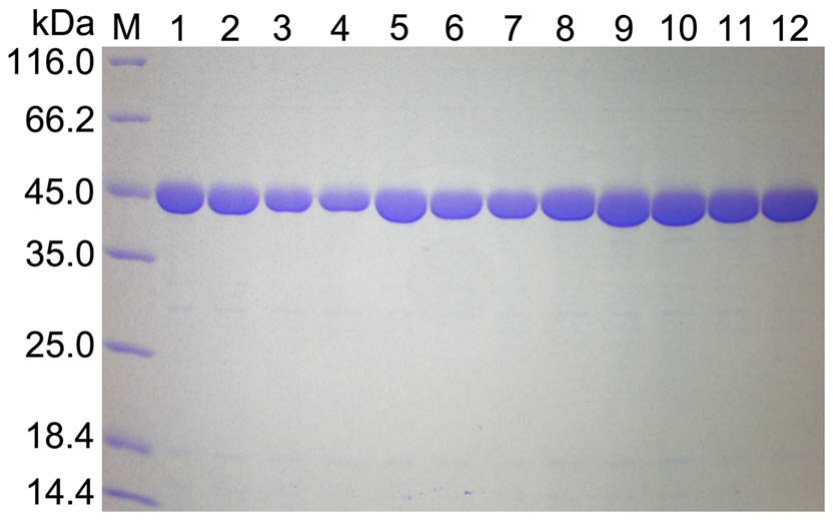
SDS-PAGE analysis of the expression and purification of wild-type and mutant YlIDH, ScIDH1 and HcIDH. Analysis was performed using a 12% polyacrylamide gel. M, protein molecular weight markers; lane 1 to 4, purified proteins of YlIDH, YlIDH R141H, YlIDH R141A and YlIDH R141E, respectively; lane 5 to 8, purified proteins of ScIDH, ScIDH R148H, ScIDH R148A and ScIDH R148E, respectively; lane 9 to 12, purified proteins of HcIDH, HcIDH R132H, HcIDH R132A and HcIDH R132E, respectively.

The mutated versions of ScIDH1, YlIDH and HcIDH were generated by the fusion-PCR-based site-directed mutagenesis method, as described above. The wild-type and mutant enzymes were expressed in *E. coli* Rosetta (DE3) cells as 6×His-tagged fusion proteins and purified by immobilized metal affinity chromatography. The SDS-PAGE gel patterns indicated that the wild-type and mutated ScIDH1, YlIDH and HcIDH were well expressed and purified to greater than 95% homogeneity (Fig. 3).

### Secondary structure determination

**Figure 4:**
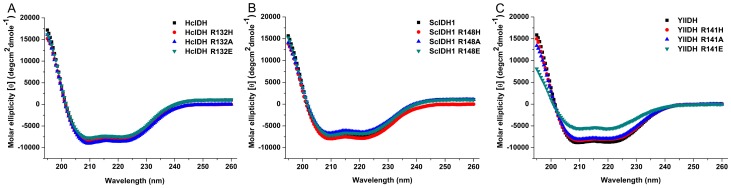
Circular dichroism (CD) spectra analysis of intact and mutant YlIDH, ScIDH1 and HcIDH. The CD was measured, and the molar ellipticity was calculated as described in the Materials and Methods. (A) The molar ellipticities of HcIDH (▪), HcIDH R148H (•), HcIDH R132A (▴) and HcIDH R132E (▾) from 195 to 260 nm; (B) the molar ellipticities of ScIDH1 (▪), ScIDH1 R148H (•), ScIDH1 R148A (▴) and ScIDH1 R148E (▾) from 195 to 260 nm; (C) the molar ellipticities of YlIDH (▪), YlIDH R141H (•), YlIDH R141A (▴) and YlIDH R148E (▾) from 195 to 260 nm.

CD spectroscopy was used to determine the secondary structure of the wild-type and single-point-mutated enzymes, and the results are shown in Fig. 4. The CD spectrum of ScIDH1 R148H, ScIDH1 R148A and ScIDH1 R148E were very similar to that of the wild-type enzyme, and similar results were obtained for YlIDH and HcIDH and their mutants. The results indicate that although the conserved Arg residue lies in the active sites of ScIDH1, YlIDH and HcIDH, its mutation does not lead to significant changes in the secondary structure of the enzymes. The YlIDH R141E mutant exhibits a small difference in its CD spectrum, which may reflect a minor change in its secondary structure.

### The isocitrate oxidation activities of wild-type and mutated enzymes

The HcIDH Arg132 mutation nearly inactivates the enzyme, leading to the loss of the ability to oxidize isocitrate to α-KG. However, the mutation provides a new reductive function of catalyzing the NADPH-dependent converting of α-KG to 2-HG [14], [15], [17], [18], [28]. As the overall structures of yeast NADP^+^-IDHs are highly homologous to those of human IDH1 and IDH2, it can be expected that an analogous mutation introduced into yeast NADP^+^-IDHs will also confer the same neomorphic α-KG reduction ability to the mutated enzyme. We attempted this conversion for two yeast NADP^+^-IDHs, one was the well-known yeast *S. cerevisiae* (ScIDH1) and the other was the “non-conventional” yeast *Y. lipolytica* (YlIDH).

The importance of Arg148, Arg141 and Arg132 to the isocitrate oxidation activity of ScIDH1, YlIDH and HcIDH, respectively, was assessed by site-directed mutagenesis in the first place. Each arginine was mutated into either the small, neutral alanine, which cannot participate in hydrogen bonding, or the large, negatively charged glutamic acid, which will repel the negatively charged isocitrate. The *K*_m_ values of ScIDH1 R148A, YlIDH R141A and HcIDH R132A were substantially increased by 216-fold, 31-fold and 270-fold, respectively (Table 2). Meanwhile, their specific activities toward isocitrate were reduced by 3-fold, 22-fold and 135-fold, respectively (Table 2). The mutation of arginine to glutamic acid caused even more severe drop in the wild-type activity, since YlIDH R141E and HcIDH R132E were completely inactivated while ScIDH R148E retained only 3% of the wild-type specific activity (Table 2). Our biochemical data showed that Arg148, Arg141 and Arg132 have pivotal roles in both isocitrate binding and catalysis in ScIDH1, YlIDH and HcIDH, respectively.

**Table 2:** Comparison of the kinetic parameters of the isocitrate oxidation activity of the wild-type and mutant IDH enzymes.

Enzyme	Isocitrate^a^			NADP^+^	Specific activity (U/mg)	Ref.
	*K*_m_ (µM)	*k*_cat_ (s^−1^)	*k*_cat_/*K*_m_ (µM^−1 ^s^−1^)	*K*_m_ (µM)		
ScIDH1	10.5±1.2	26.3±0.09	2.5	17.4±2.01	17.1±1.6	This study
ScIDH1 R148H	24.8±3.2	0.27±0.015	0.011	35.6±2.36	0.16±0.03	This study
ScIDH1 R148A	2263.1±177.3	12.4±5.31	0.005	25.7±5.09	6.1±0.06	This study
ScIDH1 R148E	1296.3±7.3	0.62±0.026	0.0005	23.2±2.60	0.57±0.03	This study
YlIDH	11.7±1.1	26.6±0.87	2.3	4.1±0.76	31.1±1.6	This study
YlIDH R141H	25.6±2.3	0.14±0.006	0.005	15.3±0.79	0.12±0.009	This study
YlIDH R141A	367.2±7.6	1.2±0.18	0.003	9.6±1.27	1.39±0.23	This study
YlIDH R141E	na	na		na	na	This study
HcIDH	5.7±1.4	15.6±0.59	2.7	11.4±2.30	18.9±0.31	This study
HcIDH R132H	2282.3±195.5	0.41±0.13	0.0002	15.8±1.67	0.15±0.006	This study
HcIDH R132A	1539.6±213.8	0.22±0.042	0.0001	18.1±2.50	0.14±0.006	This study
HcIDH R132E	na	na		na	na	This study
HcIDH	65	440,000	6,769	49		[17]
HcIDH R132H	370	37.5	0.1	84		[17]
HcIDH	57	130,000	2,281	49		[28]
HcIDH R132C	87,000	710	0.008	21		[28]
HcIDH	6.2	56.9	9.2			[16]
HcIDH R132H	582.4	31.9	0.05			[16]
HcIDH	6.39±0.55	11±0.35	1.72		14.2±0.5	[18]
HcIDH R132H	1,280±130	0.6±0.02	0.00047		0.76±0.03	[18]
HcIDH	7	12.5	1.9	4.7		[19]
HcIDH R132H	33.4	2	0.06	5		[19]

a When calculating the *K*_m_ for isocitrate, the concentration of D-isocitrate was calculated as 50% of the total DL-isocitrate in this study. “na” indicates no measurable activity.

**Figure 5:**
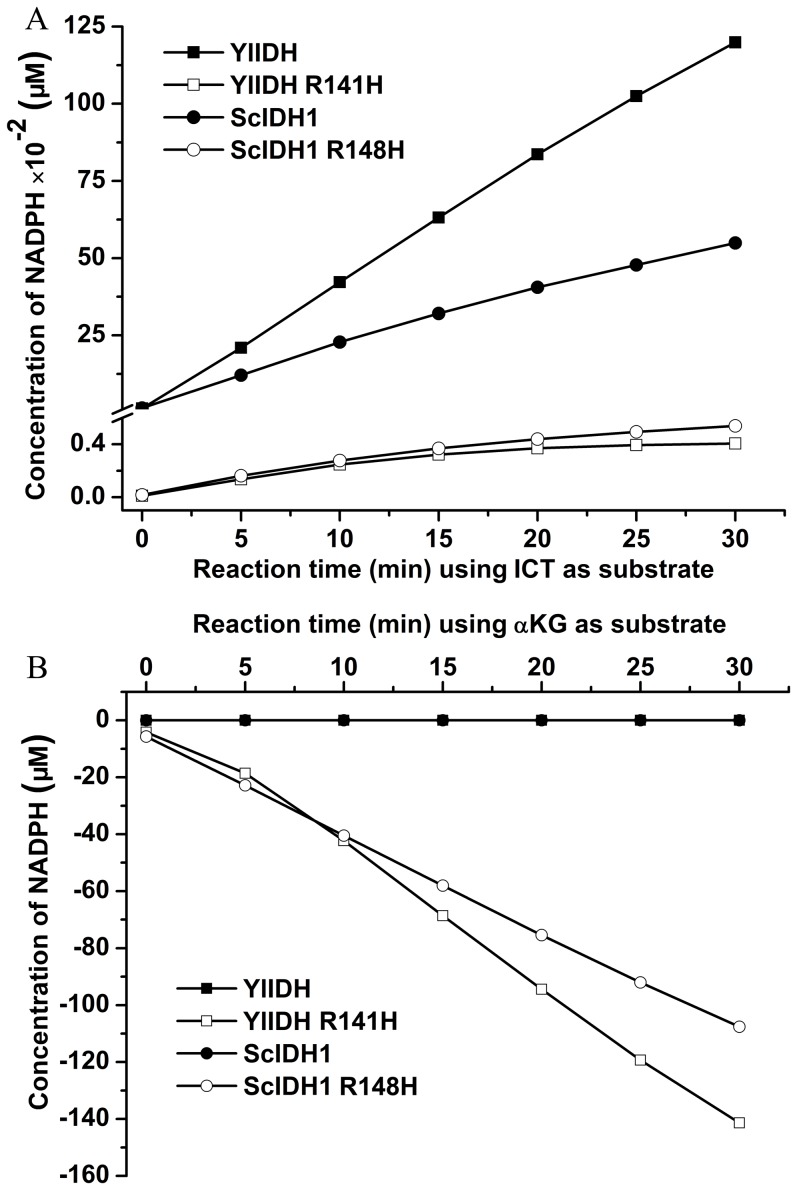
Normal and neomorphic reaction analysis of the wild-type and mutant YlIDH and ScIDH1. (A) The oxidation activity was determined using the standard assay conditions. The oxidation reaction was initiated by adding the appropriate amount of enzymes. The increasing of NADPH catalyzed by YlIDH (▪), YlIDH R141H (□), ScIDH1 (•) and ScIDH1 R148H (○), respectively. (B) The reduction of α-KG was determined using standard assay conditions. The enzymatic reaction was initiated by adding an appropriate amount of enzyme. The decrease of NADPH catalyzed by YlIDH (▪), YlIDH R141H (□), ScIDH1 (•) and ScIDH1 R148H (○), respectively.

The loss of the wild-type activities of ScIDH1 and YlIDH by the Arg mutation was shown by the slow generation of NADPH by ScIDH1 R148H and YlIDH R141H (Fig. 5A). The specific activities of ScIDH1 R148H and YlIDH R141H towards isocitrate were about 106.9-fold and 259.2-fold lower, respectively, than that of the wild-type enzyme (Table 2). The kinetic analysis showed that the *K*_m_ values of ScIDH1 R148H for isocitrate was 2.4-fold higher than that of the corresponding wild-type enzyme, whereas that of YlIDH R141H was 2.2-fold higher than that of its corresponding wild-type enzyme, suggesting that the mutated enzymes still have a relatively high affinity for isocitrate and that their impaired isocitrate catalyzing activities are not mainly caused by their inability to bind isocitrate (Table 2). However, the catalytic turnover values (*k*_cat_) of ScIDH1 R148H and YlIDH R141H toward isocitrate were severely reduced by 97-fold and 190-fold, respectively, of that of their wild-type enzymes (Table 2). The decreased *k*_cat_ values led to the largely reduced catalytic efficiencies (*k*_cat_/*K*_m_) of ScIDH1 R148H and YlIDH R141H for isocitrate oxidation, which decreased by 227-fold and 460-fold of that of wild-type enzyme (Table 2). Because Arg does not participate in cofactor binding, the affinities of ScIDH1 R148H and YlIDH R141H for NADP^+^ were only slightly affected by the mutation, as indicated by their similar *K*_m_ values (Table 2).

The enzymatic parameters of the wild-type and mutated HcIDH from five independent studies are summarized in Table 2. These results suggest that there is a large difference in the kinetic properties of wild-type HcIDH as determined by different research groups. For example, the catalytic constant (*k*_cat_) for isocitrate reported by Gross et al. [28] was more than 10,000-fold larger than that determined by Yang et al. [18]. Moreover, there are disagreements in the kinetic parameters of the HcIDH Arg132 mutants that were reported by several researchers, as the catalytic efficiency (*k*_cat_/*K*_m_) of the R132H mutant for isocitrate that was reported by Dang et al. [17] was more than 3935-fold higher than that reported by Yang et al. [18]. We also introduced the R132H mutation into HcIDH and determined the kinetic parameters of both wild type HcIDH and R132H mutant (Table 2). Our data were in the best agreement with that reported by Yang et al. [18], as the catalytic efficiency we determined for HcIDH R132H was in the same order of magnitudes with that from Yang's study (Table 2).

As shown in Table 2, all five studies confirmed that the impaired activity of HcIDH was caused by a dramatic decrease in the substrate affinity, because the mutants' *K*_m_ values for isocitrate were found to be 5-fold to 1500-fold higher than that of the wild-type enzymes. However, the analogous mutations in ScIDH1 and YlIDH did not significantly abrogate the enzymes' binding affinities for isocitrate, as demonstrated by the slightly increased *K*_m_ values (approximately 2-fold higher) for isocitrate that were displayed by the ScIDH1 R148H and YlIDH R141H mutants compared to those of the wild-type enzymes (Table 2). Despite retain the ability to bind isocitrate, the ScIDH R148H and YlIDH R141H mutants showed a severely reduced rate of converting the bound isocitrate, suggesting that effects of the ScIDH1 and YlIDH Arg mutation occurred via a different mechanism compared to that of HcIDH.

### The α-KG reduction activities of wild-type and mutant enzymes

The neoenzymatic activity of reducing α-KG to 2-HG was determined using a standard assay. Wild-type ScIDH1 and YlIDH could not catalyze this conversion, as no changes in NADPH were detected in the reaction mix (Fig. 5B). The neoenzymatic activities of ScIDH1 R148H and YlIDH R141H were clearly demonstrated by the evident NADPH-dependent consumption of α-KG (Fig. 5B). The catalytic efficiencies of the two mutants showed that ScIDH1 R148H is 1.2-fold more effective than YlIDH R141H at producing 2-HG (Table 3).

**Table 3:** Comparison of the kinetic parameters for the α-KG reduction activity of the mutant IDHs.

Enzyme	α-KG			NADPH	Specific activity (U/mg)	Ref.
	*K*_m_ (µM)	*K*_cat_ (s^−1^)	*k*_cat_/*K*_m_ (µM^−1^ s^−1^)	*K*_m_ (µM)		
ScIDH1 R148H	30.0±1.76	0.34±0.009	0.011	5.9±0.51	0.17±0.002	This study
YlIDH R141H	47.3±4.2	0.43±0.017	0.009	3.2±0.43	0.26±0.001	This study
HcIDH R132H	156.7±38.4	0.24±0.021	0.002	6.7±1.6	0.19±0.001	This study
HcIDH R132H	965	1000	1.04	0.44	-	[17]
HcIDH R132C	295	550	1.86	0.3	-	[28]

The acquisition of neomorphic activities by ScIDH1 R148H and YlIDH R141H can be explained by their emergent abilities to bind α-KG. The kinetic analysis demonstrated that ScIDH1 R148H and YlIDH R141H showed considerable affinity towards both α-KG and NADPH (Table 3). The *K*_m_ value of ScIDH1 R148H for α-KG was 32-fold lower than that of the HcIDH R132H mutant determined by Dang et al. [17]. Similarly, YlIDH R141H had a 20-fold higher affinity for α-KG than HcIDH R132H [17]. However, the *k*_cat_ values of ScIDH1 R148H and YlIDH R141H for α-KG were much smaller (approximately 0.04%) than those of the HcIDH R132H and R132C mutants reported from other studies [17], [28] (Table 3). The huge discrepancy in the α-KG reduction activity between the yeast IDH Arg mutants of this study and HcIDH R132H mutant from other groups promoted us to perform the HcIDH Arg mutation experiment in our research as well. The kinetic parameters determined for the HcIDH R132H mutant suggested that the Arg mutation in HcIDH did not confer the mutant enzyme that high activity towards α-KG as reported previously [17], [28]. HcIDH R132H mutant, by contrast, showed lower catalytic efficiency for α-KG when compared to ScIDH1 R148H (18%) and YlIDH R141H (22%) as determined by our assays (Table 3). The specific activities towards α-KG of both yeast IDH Arg mutants and HcIDH R132H mutant were proved to be very similar, being around 0.2 U/mg (Table 3).

The performance of the HcIDH R132H mutant in transforming α-KG to 2-HG was proposed to be assisted by the fine-tuned conformational adaptation of the substrate-binding site [17], [18]. The conservation of amino acid residues at the corresponding positions of ScIDH1 and YlIDH (Fig. 2), and the comparable capabilities of ScIDH1 R148H and YlIDH R141H in reducing α-KG demonstrated the similar coordinated conformation-adjusting mechanism in these two mutated yeast IDHs. HcIDH Arg132 mutation is certainly involved in the tumorigenesis, but the detailed roles that HcIDH Arg132 mutants play in cancer cell metabolism has not been clearly demonstrated due to the complexity of human cells. One implication from this study is that the metabolic pathways disturbed by the mutated IDH can be explored using yeast as model organism, because yeast is a much simpler unicellular eukaryote and its molecular interaction networks are highly similar to human cells [29], [30].

### GC/TOF-MS identification of 2-HG

**Figure 6:**
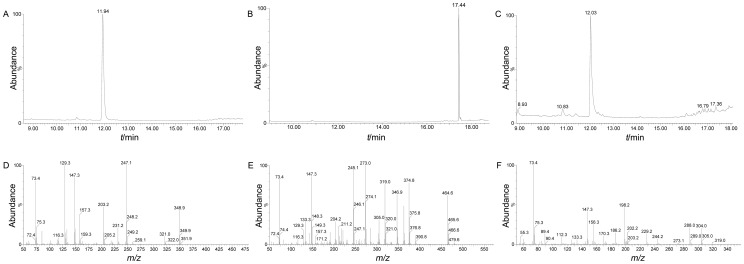
GC/TOF-MS identification of the 2-HG, ICT and α-KG standards. The retention times of the 2-HG, ICT and α-KG standards in the gas chromatogram were 11.94 min (A), 17.44 min (B) and 12.03 min (C), respectively. (D), (E) and (F) show the mass spectrogram identifications of 2-HG, ICT and α-KG, respectively.

**Figure 7:**
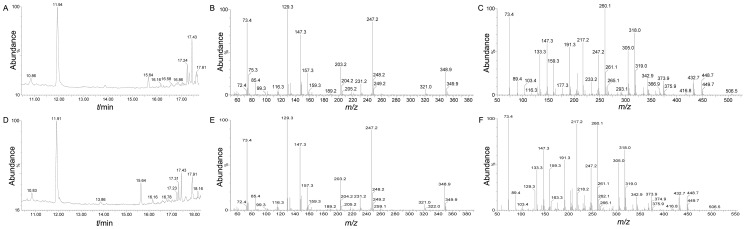
GC/TOF-MS identification of the products generated by YlIDH R141H and ScIDH1 R148H catalysis. (A) and (D) Gas chromatograms of the products generated by YlIDH R141Hand ScIDH1 R148H catalysis, respectively. (B) and (E) Mass spectrogram identifications of the 11.94-min peak in (A) and 11.91-minpeak in (D), respectively.(C) and (F) Mass spectrogram identifications of the 17.43-min peak in (A) and (D), respectively.

High-purity (>98%) 2-HG, ICT and α-KG were identified by GC/TOF-MS as standards, having retention times of 11.94 min (Fig. 6A), 17.44 min (Fig. 6B) and 12.03 min (Fig. 6C), respectively. The corresponding mass spectrogram of the three standards is shown in Figs. 6D-6F. Samples of these action mixtures catalyzed by YlIDH R141H and ScIDH R148H were then characterized in the same manner. 2-HG was identified as the end product for both the YlIDH R141H 11.94 min (Fig. 7A) and ScIDH R148H 11.91 min (Fig. 7D) mutant enzymes in the gas chromatogram, respectively. The mass spectrograms for the YlIDH R141H 11.94 min peak (Fig. 7B) and the ScIDH R 148H 11.91 min peak (Fig. 7E) were both consistent with that of the 2-HG standard (Fig. 6D), conforming the production of 2-HG from α-KG by the two mutant enzymes. Although a conspicuous ICT peak at 17.43 min was both detected in YlIDH R141H (Fig. 7A) and ScIDH R148H (Fig. 7D) in the gas chromatogram, but the corresponding mass spectrograms of YlIDH R141H 17.43 min (Fig. 7C) and ScIDH R148H 17.43 min (Fig. 7F) suggest that the both conspicuous ICT peaks were different from that of the ICT standard (Fig. 6E), demonstrating that ICT was not produced from α-KG by the residual IDH activity of the two mutant enzymes. All substrates were completely transformed into 2-HG in the dependence of NADPH in the assay, as no remaining α-KG was detected in the reaction mixture samples by GC/TOF-MS.

## Conclusions

In summary, we introduced the single point mutations Arg148His and Arg141His into ScIDH1 and YlIDH, respectively, because the analogous mutation was found in HcIDH of tumor patients. As expected, the normal isocitrate oxidation activities of the ScIDH1 R148H and YlIDH R141H mutants were nearly eliminated, and the neoenzymatic ability to catalyze the conversion of α-KG to 2-HG was acquired by the mutants, and the catalytic activities of ScIDH1 R148H and YlIDH R141H for α-KG were comparable to that of the HcIDH R132H mutant. Since the HcIDH Arg132 mutation promotes tumorigenesis in human cells, a parallel role of the Arg mutated IDHs in yeast cells deserves in-depth exploration in the future.
